# The application of standardised diagnostic criteria in RCTS in depression

**DOI:** 10.1186/1745-6215-14-S1-P72

**Published:** 2013-11-29

**Authors:** Finola Ferry, Gillian Shorter, Mike Clarke

**Affiliations:** 1 University of Ulster, Londonderry, UK; 2 MRC All Ireland Methodology Hub, Belfast, UK

## Introduction

Just as RCTs represent the standard of testing interventions in healthcare, the use of standardised diagnostic classification systems ICD and DSM, represent a key standard in mental health research. Despite this, the application of these classification systems in RCTs in mental health has yet to be fully explored.

## Methods

A systematic review was undertaken of RCTs in depression examining trials conducted from the year 2000 registered in the Cochrane Reviews Depression, Anxiety and Neurosis Group. The review, still in progress, aims to identify the measurement instruments and methods used to identify depression outcomes and whether these are aligned with standardised diagnostic criteria for depression.

## Results

Early results from completed trials show considerable variation in the methods and instruments used to assess depression. The majority of studies have used symptom based instruments including the Hamilton Rating Scale for Depression, the Beck Depression Inventory, the Global Clinical Improvement Scale and Hospital Anxiety and Depression Scale, while other trials reported the use of clinical assessment without specification of a particular instrument. Despite the reported use of DSM or ICD criteria as inclusion criteria in many of these trials, these classification systems have been applied to a much lesser extent in the measurement of depression outcomes.

## Conclusion

Evidence suggests that ICD and DSM criteria for depression are not routinely applied in RCTs, with considerable variation in the use of instruments and effect measurement. Wider application of these gold standard classification systems would help improve comparability of outcomes in Psychiatry and Mental Health trials.

